# HALP-H Index as a Prognostic Biomarker for Predicting Pathological Complete Response in Early-Stage HER2-Positive Breast Cancer—A Multicenter Retrospective Cohort Study

**DOI:** 10.3390/jcm14134431

**Published:** 2025-06-22

**Authors:** Mustafa Seyyar, Pervin Can Şancı, Tolga Köşeci, Anıl Karakayalı, Mutianur Özkorkmaz Akdağ, Yasemin Bakkal Temi, Kazım Uygun, Umut Kefeli, Burak Mete, Devrim Çabuk

**Affiliations:** 1 Departmant of Medical Oncology, Gaziantep City Hospital, Gaziantep 27100, Türkiye; mustafa.seyyar@saglik.gov.tr; 2 Department of Medical Oncology, Kocaeli City Hospital, Kocaeli 41000, Türkiye; pervin.cansanci@saglik.gov.tr (P.C.Ş.); anil.karakayali@saglik.gov.tr (A.K.); 3 Department of Medical Oncology, Faculty of Medicine, Çukurova University, Adana 01250, Türkiye; tkoseci@cu.edu.tr; 4 Department of Medical Oncology, Faculty of Medicine, Kocaeli University, Kocaeli 41380, Türkiye; mutianur.ozkorkmaz@kocaeli.edu.tr (M.Ö.A.); yasemin.temi@kocaeli.edu.tr (Y.B.T.); kazim.uygun@kocaeli.edu.tr (K.U.); devrim.cabuk@kocaeli.edu.tr (D.Ç.); 5 Department of Public Health, Faculty of Medicine, Çukurova University, Adana 01250, Türkiye

**Keywords:** pathological response, HALP-H index, neoadjuvant treatment, HER2-positive breast cancer

## Abstract

**Objectives:** Inflammation plays an important role in cancer development, and various inflammation parameters are used as potential prognostic markers. This study aimed to evaluate the effectiveness of the combined use of HALP and H index in predicting pathological response to neoadjuvant therapy in patients with HER2-positive early-stage breast cancer. **Method:** This retrospective cohort study was conducted on 146 HER2-positive breast cancer patients treated in two centers. To stratify patients by their predicted probability of pathological response, HALP and H index values were combined into a composite biomarker score called the combined response score (CRS). Patients were classified into three groups based on biomarker levels: 0 = low CRS (low predictive score), 1 = intermediate CRS, and 2 = high CRS (high predictive score). These groups reflect predicted response likelihood and do not represent actual pathological outcomes. Pathological response results were evaluated according to the combined response score. Pathological complete response (pCR) was defined as residual cancer burden (RCB) 0, indicating no residual invasive tumor in breast or lymph nodes. **Results:** The mean age of 146 early-stage breast cancer patients included in our study was 52.3 ± 11.3 (min: 29-max: 83). In the ROC analysis, the optimum cut-off value for the HALP score in pathological response classification was found to be 36 (AUC = 0.608, sensitivity = 76.29%, specificity = 44.9%, PPV = 73%, NPV = 47.89%) and 2.3 for the H index (AUC = 0.641, sensitivity = 65.98%, specificity = 51.02%, PPV = 72.73%, NPV = 43.1%). While the pathological complete response rate was 66.4% in all patients, it was 81.8% in those with a combined score of 2, 51% in those with a score of 1, and 58.6% in those with a score of 0 (*p* < 0.001). In the logistic regression analysis, the probability of pathological response in patients in the combined score = 2 group is 3.77 times higher than in group 0. In the Fagan nomogram, the pretest probability of pathological response is 66%, while the post-test probability for combined response score group 2 is 81.5%, and for the low-H index < 2.3 and the high-HALP ≥ 36 patient group, our estimate for pathological complete response increases to 82%. **Conclusions:** The HALP-H index combined score is an important predictor of pathological response in early-stage HER2-positive breast cancer patients, independent of histological type and stage. This new score may enable personalized approaches in treatment planning.

## 1. Introduction

Breast cancer is the most prevalent malignancy among women globally and constitutes a significant cause of cancer-related mortality. Breast cancer exhibits marked heterogeneity across clinical presentation, histopathological features, and molecular profiles, which profoundly influences therapeutic response and disease prognosis [1]. The HER2-positive subtype, including roughly 20–25% of breast tumors, is linked to an aggressive disease trajectory and unfavorable prognosis [2]. However, HER2-targeted therapies developed in recent years have significantly increased survival rates in this subtype. Neoadjuvant therapy (NAT) is an increasingly used treatment method for HER2-positive early-stage breast cancer [3]. The NAT approach has emerged as a standard treatment strategy, particularly for HER2-positive breast tumors, with a pathological complete response (pCR) regarded as a favorable prognostic sign for long-term survival [4]. A wide range of clinical trials have explored emerging HER2-targeted treatment strategies aimed at enhancing pCR rates in NATs. While the addition of trastuzumab to neoadjuvant chemotherapy increased pCR rates, the highest pCR rates were achieved when pertuzumab was added to trastuzumab [5,6,7]. However, not all patients respond equally to NAT. Therefore, identifying biomarkers that can predict response to NAC is of great importance for personalizing treatment strategies and preventing unnecessary toxicities [8]. Despite ongoing efforts to develop biomarkers for predicting therapeutic response to neoadjuvant chemotherapy with trastuzumab and pertuzumab, clarity remains elusive. These agents are monoclonal antibodies that attach to the extracellular subdomain of the HER2 receptor on cancer cell surfaces. Furthermore, trastuzumab and pertuzumab induce antibody-dependent cell-mediated cytotoxicity, indicating that immune activity within the tumor microenvironment may influence treatment effectiveness [9]. Elevated baseline levels of tumor-infiltrating lymphocytes (TILs) have been correlated with higher rates of pCR in HER2-positive breast cancer patients undergoing neoadjuvant HER2-targeted therapy. Nevertheless, the evaluation of TILs or other tissue-based immune-related metrics has not been consistently conducted in clinical practice. Consequently, there is a want for more accessible immunological markers that can predict neoadjuvant response in HER2-positive breast cancer. Given the pivotal role of inflammation and immune mechanisms in tumorigenesis, a range of inflammatory markers and composite indices have been explored as potential prognostic indicators across multiple cancer types. Combining these indices usually provides a more accurate prognosis than a single parameter, and they can be derived from routine laboratory tests [10]. Studies on the role of inflammation in HER2-positive breast cancer emphasize the prognostic importance of inflammatory markers as well as their value in predicting response to treatment. In particular, the prognostic values of markers such as the neutrophil–lymphocyte ratio, platelet–lymphocyte ratio, and systemic immune-inflammation index have been shown in various studies [8,11,12]. The hemoglobin, albumin, lymphocyte, and platelet (HALP) score, which integrates indicators of inflammation, nutritional status, and immune function, was initially introduced by Chen et al. in 2015 as a prognostic tool for survival outcomes in patients with gastric cancer [13]. In recent years, the HALP score has been utilized across numerous malignancies as a predictive marker for treatment response and therapeutic outcomes [14,15]. A recently identified biomarker is the host index (H-Index), which was initially investigated by Valero et al. in 2020 in patients with oral cavity squamous cell carcinoma undergoing primary surgical treatment [16]. This novel composite indicator was established by amalgamating neutrophil, monocyte, lymphocyte, albumin, and hemoglobin levels. However, there is no known study evaluating the effectiveness of these indexes in forecasting pCR in HER2-positive breast cancer. This study aims to assess the efficacy of the HALP-H index, which combines the HALP score and the H index, in forecasting pathological response to neoadjuvant treatment in HER2-positive early and locally advanced-stage breast cancer patients.

## 2. Materials and Methods

### 2.1. Research Type and Ethics

This multicenter retrospective cohort study was conducted on patients with early and locally advanced breast cancer who were treated with neoadjuvant therapy and who applied to Gaziantep City Hospital and Kocaeli University Medical Oncology Department between 2015 and 2023. This research obtained ethical approval from the Kocaeli University Ethics Committee with the number GOKAEK-2024/20.18 (Project code: 2024/493).

### 2.2. Determination and Selection of Sample Size

**Figure 1:**
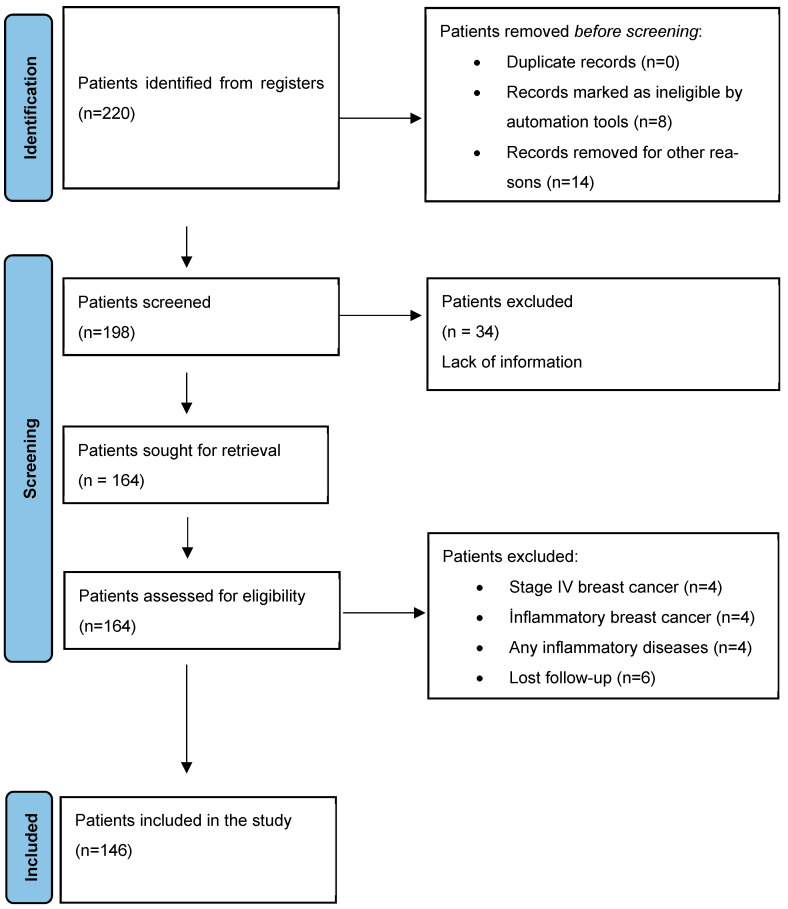
Flow chart of identification of patients via registers.

In the sample size analysis where type 1 error was accepted as 0.05, power was 90%, and effect size was d = 0.85, the minimum sample size to be reached was found to be 140. Since there was no study to refer to in the literature, the effect size was accepted as medium effect size (0.5) according to Cohen’s guidelines. A total of 220 early-stage breast cancer patients applied to the two participating centers during the study period. Among them, 8 were excluded by automated eligibility tools (e.g., duplicates, incomplete identifiers), and 14 were excluded due to administrative or technical issues (e.g., missing baseline data, unavailable lab results), resulting in 198 patients eligible for formal screening. Of these, 34 patients were excluded due to lack of medical information, and an additional 18 patients were excluded for clinical reasons (e.g., inflammatory breast cancer, metastatic disease, loss to follow-up, or concurrent inflammatory conditions). Consequently, 146 patients who satisfied all qualifying criteria were incorporated into the final study (Figure 1).

- **Inclusion criteria:**
  - Patients ≥ 18 years old diagnosed with invasive breast cancer;
  - Patients without metastasis;
  - Patients receiving neoadjuvant chemotherapy containing trastuzumab + pertuzumab in early or locally advanced stages (specifically, TNM stage IIA to IIIC based on AJCC 8th edition).

- **Exclusion criteria:**
  - Individuals with organ failure;
  - Individuals whose performance score is not suitable for chemotherapy treatment;
  - Individuals with autoimmune diseases;
  - Individuals with secondary malignancies;
  - Individuals afflicted by inflammatory illnesses.

All patients underwent 4 cycles of dose-dense doxorubicin + cyclophosphamide and 4 cycles of docetaxel + trastuzumab + pertuzumab as neoadjuvant treatment. Doxorubicin (60 mg/m^2^, 15 min infusion) and cyclophosphamide (600 mg/m^2^, 30 min infusion) were administered every 2 weeks for 4 cycles. This was followed by docetaxel (75 mg/m^2^, 60-min infusion), trastuzumab (8 mg/kg loading dose, then 6 mg/kg in 30 min), and pertuzumab (840 mg loading dose, then 420 mg in 30–60 min), all given every 3 weeks for 4 cycles.

Patients’ age, hemoglobin, neutrophil, neutrophil, lymphocyte, lymphocyte, platelet, monocyte values, liver and kidney function tests, albumin levels, treatment start date, death date, progression date, and pathology findings were extracted from their electronic medical records.

### 2.3. Pathological Assessment

Resected breast tissue specimens were preserved in formaldehyde and subsequently embedded in paraffin blocks. Histological sections were then prepared and stained using hematoxylin and eosin (H&E) for microscopic evaluation. The pCR was defined as residual cancer burden (RCB) 0, indicating no residual invasive tumor in the breast or lymph nodes. Pathological response status was determined according to the RCB scoring system from the pathology specimen obtained after surgery [17]. The calculation of the RCB score was performed by two pathologists based on the RCB evaluation system. Pathologists reanalyzed and classified patients’ pathological data using the RCB calculator provided by MD Anderson Cancer Center (www.mdanderson.org/breastCancer_RCB (accessed on 15 April 2025)) [5]. According to the RCB system, patients are categorized into four different classes:

- RCB 0: pCR indicates no residual tumor, and RCB score is 0.
- RCB I: Minimal residual burden, RCB score greater than 0 and less than or equal to 1.36.
- RCB II: Moderate residual burden, RCB score greater than 1.36 and less than or equal to 3.28.
- RCB III: Extensive residual burden, with an RCB score greater than 3.28.

### 2.4. Combine Response Score Calculating

The combined response score (CRS) used in this study is a predictive stratification based on HALP and H-index levels and does not redefine pCR or RCB-based outcomes. Before starting targeted therapy, patients’ albumin and hemoglobin levels and platelet, neutrophil, monocyte, and lymphocyte counts were determined, followed by calculation of the HALP score [13] and H index [16] as shown below:

$$HALP\;score=\frac{{Haemoglobin\left(g/L\right)\times Albumin\left(g/L\right)\times Lymphocyte\;count(L}^{-1})}{Platelet\;count{(L}^{-1})}$$

**H-index** = (neutrophil count × monocyte count)/(Hb × albumin × lymphocyte count) × 100

**Figure 2:**
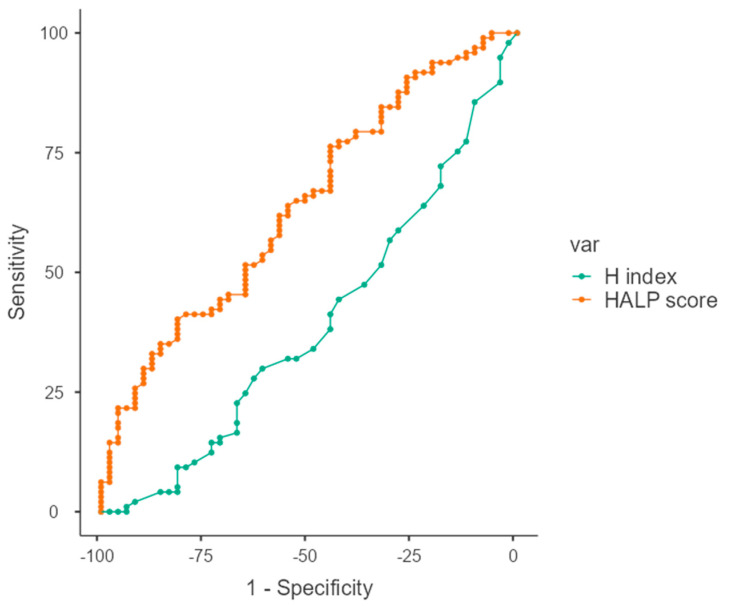
ROC curves of HALP score and H index in predicting pathological complete response (pCR), showing respective AUC values and optimal cut-off points.

The cut-off value was determined by receiver operating characteristics (ROC) analysis. The cut-off was found to be 36 (AUC = 0.608) for the HALP score and 2.3 (AUC = 0.641, *p* < 0.001) for the H index (Figure 2).

The patients were grouped based on the cut-off value according to the ROC analysis results: “low response-HALP group” if the AUC was ≤36 and “high response-HALP group” if the AUC was >36; “high response-H index group” if the AUC was ≤2.3; and “low response-H index group” if it was >2.3. We combined HALP and H index values into a composite biomarker score called the CRS and stratified patients into three predictive groups. Patients with both high H index (>2.3) and low HALP score (<36) were classified as the “low CRS group” (indicating a low predicted probability of pCR); patients with either high H index or low HALP score were assigned to the “moderate CRS group”; and those with both low H index (≤2.3) and high HALP score (≥36) were classified as the “high CRS group” (indicating a high predicted probability of pCR). These CRS groups are based solely on biomarker levels and do not reflect actual pathological outcomes (Table 1).

### 2.5. Statistical Analysis

Statistical analyses were performed using JAMOVI version 2.6.17 (The Jamovi Project, www.jamovi.org; accessed on 1 October 2024). Data normality was evaluated using the Kolmogorov–Smirnov test and by visually inspecting probability plots. Categorical variables were compared using Pearson’s chi-square test or Fisher’s exact test, depending on appropriateness. Continuous variables were analyzed with one-way ANOVA when normally distributed and with the Kruskal–Wallis test when normality assumptions were not met. ROC curve analysis, along with the Youden index, was employed to identify optimal cut-off values for the HALP and H indices in predicting pCR. Variables with a *p*-value < 0.10 in univariate logistic regression were selected for inclusion in the multivariate logistic regression model, which was constructed using a backward stepwise elimination approach. The final model retained variables with statistical significance at *p* < 0.05. The following variables were included in the multivariate model: age, tumor size, hormone receptor status, histological grade, HALP score, and H index. In addition, Fagan nomogram analysis was conducted to calculate post-test probabilities of pCR based on sensitivity and specificity values derived from ROC analysis. Positive and negative likelihood ratios (LR+ and LR−) were used to estimate post-test response and non-response probabilities. A *p*-value < 0.05 was considered statistically significant.

## 3. Results

The average age of the 146 breast cancer patients in our study was 52.3 ± 11.3 years (min:29–max:83). While the pathologic complete response rate was 66.4% in the whole group, it was 81.8% in patients with a combined score of 2, 51% in patients with a combined score of 1, and 58.6% in patients with a combined score of 0. When the demographic and clinical characteristics of the patients were compared according to the combined score grouping, it was found that there were no significant differences in age, Ki67, ER%, PR%, number of lymph nodes, comorbidity, histology, stage, menopausal status, treatment options, and only in localization, left localization was significantly different in the intermediate response group (Table 2).

Logistic regression analysis to predict pathologic response to neoadjuvant treatment was found to be significant (omnibus test *p* = 0.012). The goodness of fit of the model was found to be adequate with Nagelkarke R square = 0.302, accuracy rate 72.4%, and sensitivity 86.5%. Among the independent variables included in the model, combined response score and ER positivity were found to make a significant contribution. In the combined score, the probability of pathologic response was 3.77 times higher in patients in the high response group and 0.299 times (OR = 3.44) lower in patients with ER positivity (Table 3).

**Figure 3:**
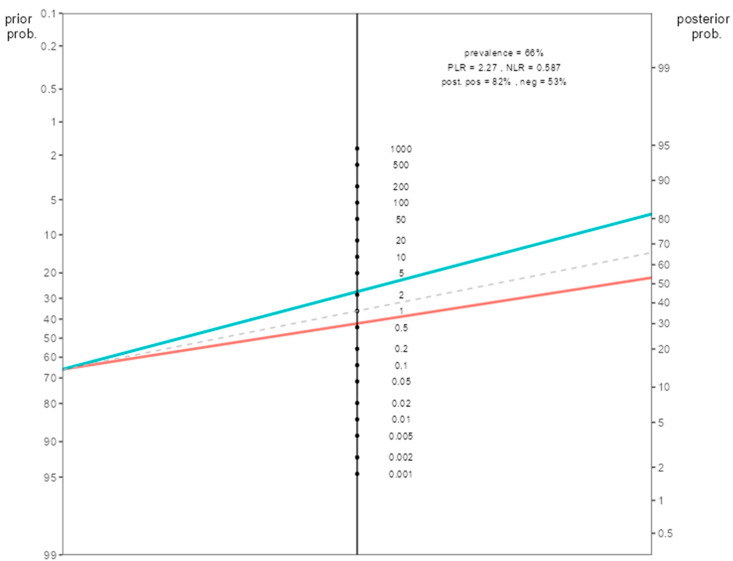
Fagan nomogram illustrating the post-test probability of pathological complete response (pCR) in patients with high combined response score (CRS = 2), based on sensitivity and specificity derived from ROC analysis. **Note:** (The green upper line passes over the positive likelihood value, the red lower line passes over the negative likelihood value).

When the post-test probability rates were calculated for the combined response score group in the Fagan nomogram, the post-test probability of pathologic response was 81.5% in patients with a combined response score of 2. In other words, while the pretest probability of pathologic response was 66%, our post-test probability was 81.5%. For this patient group, our initial prediction for pathologic response for the low-H index < 2.3 and the high-HALP ≥ 36 patient group increases from 66% to 82% (Figure 3).

## 4. Discussion

In recent years, the importance of inflammatory indices in cancer prognosis has been increasingly emphasized. Multiple stages of tumor development are modulated by systemic inflammation, nutritional balance, and the integrity of the host immune response [18]. These factors not only provide indirect insight into cancer initiation and progression but also serve as surrogate indicators of treatment responsiveness and overall survival in clinical settings. The preoperative neutrophil-to-lymphocyte ratio index has demonstrated a diminished predictive capacity for overall survival (OS) and progression-free survival (PFS) in patients with advanced cancers [19]. A considerable number of cancer patients experience malnutrition due to the direct physiological impacts of tumors, resulting in issues such as dyspepsia, diarrhea, and the side effects of anti-tumor therapies [20]. Nonetheless, the majority of clinical research is constrained to examining the influence of a singular indicator on the incidence, progression, therapy, and prognosis of patients with malignancies. As research advances, investigators are more focused on employing comprehensive indicators to forecast the prognosis and treatment strategies for patients with cancers.

The HALP score reflects systemic inflammation and nutritional status by combining hemoglobin, albumin, lymphocyte, and platelet levels, while the H index is calculated by neutrophil, monocyte, hemoglobin, albumin, and lymphocyte levels and helps us to assess the impact of inflammatory cells from a broader perspective. The combination of these two parameters more comprehensively reflects the complex interactions between inflammation and immune response in the tumor microenvironment. The cancer microenvironment includes inflammation and immune response processes that play an important role in tumor growth and metastasis. In this context, the HALP score and H index have been studied in many cancer types as an indicator of systemic inflammation. For example, a low HALP score has been correlated to unfavorable prognostic outcomes in patients with gastric cancer [13]. Similarly, in colorectal cancer patients, the HALP score has been defined as an effective parameter in predicting survival [21]. Valero et al. [16] evaluated the H index in oral cavity cancer and reported that a high H index was associated with poor prognosis. However, the combined use of these indices in HER2-positive breast cancer and their efficacy in predicting response to neoadjuvant treatment were evaluated for the first time in this study.

In this study, the role of the combination of HALP score and H index in predicting pathological response to neoadjuvant therapy in patients with HER2-positive breast cancer was investigated. Our findings revealed that the combined use of these two inflammatory markers had a stronger predictive value than using them alone. The 3.77-fold increase in the probability of pathological response, especially in the high response group (OR = 3.77, 95% CI: 1.259–11.312, *p* = 0.018), reveals the importance of this combination in terms of clinical use. A key finding of our study is the identification of the HALP-H index as an independent predictor of pCR in HER2-positive breast cancer. Patients with a high HALP-H index exhibited significantly elevated pCR rates. In addition, Fagan nomogram results support the potential use of the HALP-H index in clinical practice. The increase in pCR probability from 66% to 81.5% in patients with high HALP-H index values emphasizes the prognostic accuracy of this scoring system.

Although previous studies have shown that HALP and H indexes have prognostic value individually [13,16], the superiority of the HALP-H index, which is the combination of these two parameters, in predicting pCR is one of the most important findings of this study. In our study, the significantly higher pCR rates in patients with high HALP-H index (OR = 3.77) support the prognostic power of this combination.

An important finding of our study is that the combined inflammatory scoring system is easy to use and cost-effective in clinical practice. HALP-H index is a biomarker with high applicability in clinical practice because it is based on easily measurable hematological parameters. Evaluation of the HALP-H index before neoadjuvant treatment may allow personalized approaches in treatment planning by estimating the probability of pCR. For example, more aggressive treatment strategies or additional therapeutic approaches can be evaluated in patients with low HALP-H index. In addition, the HALP-H index has the potential to be used as a prognostic marker in other breast cancer subtypes and different cancer types.

The findings of this study are generally consistent with the previously published literature, although slight variations were noted. In the study conducted by Chen et al. [13], which examined the prognostic significance of the HALP score in gastric cancer, a low HALP score was found to be associated with poor prognosis. Our study found that a low HALP score correlates with a diminished response to neoadjuvant therapy. Wang et al. [8] explored the predictive value of systemic inflammation markers in assessing response to neoadjuvant therapy in patients with HER2-positive breast cancer and found a significant association between the HALP score and pCR. Consistently, our findings demonstrated that patients with higher HALP scores had an increased likelihood of achieving a pathological response. This similarity suggests that the HALP score can be used as a general prognostic marker in different types of cancer. In the study by Boscolo-Rizzo et al. [22] evaluating the H index in laryngeal squamous cell carcinoma treated with surgery, it was reported that a high H index had worse 5-year OS and DFS compared to a low H index. In our study, a low H index was associated with a high pathological response. This similarity shows that the H index can be used as an inflammation-based prognostic marker in different tumor types.

Previous studies have also shown the prognostic value of inflammatory markers in breast cancer. Xue et al. [23] reported that a high neutrophil/lymphocyte ratio was associated with low pCR rates. Zhang et al. [24] also showed that the systemic immune-inflammation index was effective in predicting neoadjuvant treatment response. In our study, the combination of the HALP score and the H index showed a higher predictive value; this supports the importance of evaluating inflammatory parameters together.

In our subgroup analyses, pCR rates were found to be significantly lower in ER-positive patients (OR = 0.299, *p* = 0.040). It has also been reported in the literature that ER-positive patients respond less to neoadjuvant therapy. In the CALGB 40601 study by Carey et al. [25], the pCR rate was found to be around 70% in ER-negative HER2-positive patients, while this rate remained around 40% in ER-positive patients. Similarly, Wang et al. [8] reported higher pathological response rates in ER-negative patients. Prat et al. [26] reported that molecular subtypes in HER2-positive breast cancer lead to different survival outcomes, underlining the differences between ER-positive and ER-negative subgroups. This finding can be explained by the fact that ER-positive tumors generally have lower proliferative activity and, therefore, respond less to neoadjuvant therapy. In addition, it should not be forgotten that hormonal therapies should also be included in the treatment process in ER-positive patients. This situation once again reveals the heterogeneous structure of HER2-positive breast cancer and that the response to treatment depends not only on the HER2 status but also on the hormonal receptor status.

### Strengths and Limitations

Our study findings are consistent with previous studies in the literature but offer several unique contributions. First, the combined use of HALP and H index provided higher accuracy in predicting pCR in HER2-positive breast cancer. This suggests that combining inflammatory indices may increase prognostic power. Second, the fact that the HALP-H index is associated with pCR independently of other prognostic factors, such as ER status, increases the potential of this scoring system in clinical applications. The most important limitation of this study is that it has a retrospective design, which may have led to information bias. The small number of centers and the relatively limited number of patients may have led to selection bias.

## 5. Conclusions

The results of this study show that the combined HALP-H index is an effective biomarker in predicting pathological response to neoadjuvant therapy in HER2-positive early-stage breast cancer. The results of our study once again emphasize the importance of inflammation and immune response in cancer prognosis. The HALP-H index is a parameter that can be easily obtained and applied in clinical practice and may allow for personalized approaches in treatment planning. More comprehensive and prospective studies to be conducted in the future will further strengthen the clinical use of this index.

## Figures and Tables

**Table 1:** Assessment of combined response score.

	H Index	
HALP	≤2.3	>2.3
≥36	2	1
<36	1	0

**Table 2:** Comparison of demographic and clinical characteristics according to the combined score group.

	Combine Response Score GroupsMean ± S.D., *n* (%), Median [IQR]			
	Low Response (n: 29)	Modarate Response (n: 51)	High Response (n: 66)	*p*
**Age**	49.89 ± 12.64	50.54 ± 11.28	54.65 ± 10.40	0.070
**Ki67 %**	21.13 ± 10.83	26.09 ± 16.49	22.34 ± 10.81	0.252
**ER %**	60 [90]	40 [95]	40 [95]	0.967
**PR %**	5 [80]	1 [45]	1.5 [58.8]	0.607
**Lymph node count**	0 [1]	0 [1]	0 [0]	0.059
**Comorbidity**				
Absent	22 (78.6)	37 (72.5)	52 (78.8)	0.704
Present	6 (21.4)	14 (27.5)	14 (21.2)	
**Menopause**				
Premenapause	16 (55.2)	26 (51.0)	23 (34.8)	0.178
Postmenopause	11 (37.9)	21 (41.2)	40 (60.6)	
Perimenopause	2 (6.9)	4 (7.8)	3 (4.5)	
**Histology**				
Ductal	23 (79.3)	36 (70.6)	54 (81.8)	0.536
Mikst	6 (20.7)	13 (25.5)	11 (16.7)	
Other	0 (0)	2 (3.9)	1 (1.5)	
**Localization**				
Right	17 (58.6)	17 (33.3)	37 (56.1)	**0.021**
Left	12 (41.4)	31 (60.8)	29 (43.9)	
Bilateral	0	3 (5.9)	0	
**Grade**				
1	4 (13.8)	7 (13.7)	11 (16.7)	0.887
2	18 (62.1)	29 (56.9)	41 (62.1)	
3	7 (24.1)	15 (29.4)	14 (21.2)	
**ER status**				
Positive	18 (62.1)	31 (60.8)	37 (56.1)	0.813
Negative	11 (37.9)	20 (39.2)	29 (43.9)	
**PR status**				
Positive	17 (58.6)	26 (51.0)	33 (50.0)	0.728
Negative	12 (41.4)	25 (49.0)	33 (50.0)	
**Stage**				
1	0	0	1 (1.5)	0.789
2A	5 (17.2)	11 (21.6)	21 (31.8)	
2B	9 (31.0)	16 (31.4)	18 (27.3)	
3A	10 (34.5)	18 (35.3)	18 (27.3)	
3B	1 (3.4)	0	2 (3.0)	
3C	4 (13.8)	6 (11.8)	6 (9.1)	
**Type of surgery**				
MRM + ALND	9 (31.0)	15 (29.4)	13 (19.7)	0.171
MRM + SLND	5 (17.2)	18 (35.3)	30 (45.5)	
BCS + ALND	6 (20.7)	4 (7.8)	8 (12.1)	
BCS + SLND	9 (31.0)	14 (27.5)	15 (22.7)	
**Adjuvan CT**				
Trastuzumab	22 (75.9)	38 (74.5)	58 (89.2)	0.090
T-DM1	7 (24.1)	13 (25.5)	7 (10.8)	
**Adjuvant hormone therapy**				
Absent	8 (27.8)	14 (28.0)	22 (33.3)	0.217
Tamoxifen	14 (48.3)	25 (50.0)	22 (33.3)	
Anastrazole	3 (10.3)	9 (18.0)	18 (27.3)	
Letrozole	4 (13.8)	2 (4.0)	4 (6.1)	
**Adjuvant LHRH**				
Absent	5 (17.2)	3 (5.9)	3 (4.5)	0.083
Present	24 (82.8)	48 (94.1)	63 (95.5)	
**Pathological complete response**				
Absent	17 (58.6)	26 (51.0)	54 (81.8)	**<0.001**
Present	12 (41.4)	25 (49.0)	12 (18.2)	

**Table 3:** Logistic regression analysis predicting pathologic response.

				95% Confidence Interval	
Predictors	β	*p*	O.R.	Lower	Upper
**Intercept**	−0.6545	0.787	0.520	0.00452	59.711
**Combine response score**					
Modarate–low	−0.1276	0.810	0.880	0.31163	2.486
High–low	1.3281	**0.018**	**3.774**	1.25904	11.312
**Age**	0.0298	0.430	1.030	0.95678	1.109
**Ki67 %**	−0.0252	0.149	0.975	0.94223	1.009
**Comorbidity**					
Present-Absent	0.4168	0.483	1.517	0.47281	4.868
**ER Status**					
Positive–Negative	−1.2078	**0.040**	**0.299**	0.09464	0.944
**PR Status**					
Positive–Negative	0.5416	0.355	1.719	0.54518	5.418
**Grade**					
Grade II–Grade I	−0.1635	0.799	0.849	0.24174	2.983
Grade III–Grade I	0.0755	0.917	1.078	0.25949	4.482
**Histoloji**					
Ductal–Other	0.8907	0.559	2.437	0.12306	48.259
Ductal-Lobular–Other	0.8553	0.590	2.352	0.10449	52.942
**Menopausal Status**					
Postmenopause–Premenopause	−0.4955	0.537	0.609	0.12635	2.938
Perimenopause–Premenopause	0.5351	0.619	1.708	0.20668	14.108
**HER2 Status**					
2 positive FISH (+)–3 positive	0.1050	0.869	1.111	0.31925	3.864
**Stage**					
Stage I–Stage IIIC	14.4095	0.992	1.81 × 10^6^	0.00000	Inf
Stage IIA–Stage IIIC	0.1860	0.812	1.204	0.26005	5.578
Stage IIB–Stage IIIC	0.1633	0.824	1.177	0.28010	4.950
Stage IIIA–Stage IIIC	−1.1687	0.107	0.311	0.07498	1.288
Stage IIIB–Stage IIIC	−0.8691	0.574	0.419	0.02033	8.651

O.R.:Odds Ratio, Inf: internal fault.

## Data Availability

No new data were created or analyzed in this study.
